# Adulteration of Weight Loss Supplements by the Illegal Addition of Synthetic Pharmaceuticals

**DOI:** 10.3390/molecules26226903

**Published:** 2021-11-16

**Authors:** Ammar A. Jairoun, Sabaa Saleh Al-Hemyari, Moyad Shahwan, Sa’ed H. Zyoud

**Affiliations:** 1 Discipline of Social and Administrative Pharmacy, School of Pharmaceutical Sciences, University Sains Malaysia, Pulau Pinang 1800, Malaysia; sabaa.alhemyari@mohap.gov.ae; 2 Health and Safety Department, Dubai Municipality, Dubai P.O. Box 67, United Arab Emirates; 3 Pharmacy Department, Emirates Health Services Establishment, Dubai P.O. Box 1853, United Arab Emirates; 4 Department of Clinical Sciences, College of Pharmacy and Health Sciences, Ajman University, Ajman P.O. Box 346, United Arab Emirates; m.shahwan@ajman.ac.ae; 5 Center of Medical and Bio-Allied Health Sciences Research, Ajman University, Ajman P.O. Box 346, United Arab Emirates; 6 Poison Control and Drug Information Center (PCDIC), College of Medicine and Health Sciences, An-Najah National University, Nablus 44839, Palestine; saedzyoud@najah.edu; 7 Clinical Research Centre, An-Najah National University Hospital, Nablus 44839, Palestine

**Keywords:** adulteration weight loss supplements, undeclared pharmaceutical chemicals, adulteration behaviors, fluoxetine, phenolphthalein, sibutramine

## Abstract

Weight loss supplements that have illegal additives of pharmaceutical drugs or analogues have additional health risks, and customers may not be aware of what they are taking. This research is an essential investigation and quantification of illegally added pharmaceuticals or prescription medications, specifically fluoxetine, phenolphthalein, and sibutramine, in herbal weight loss supplements offered for sale in the United Arab Emirates (UAE). In this case, 137 weight loss supplements were collected and analyzed in this study. Reversed-phase high-performance liquid chromatography with UV absorption detection coupled to tandem mass spectrometry (RP-HPLC-MS/MS) analyses were used to determine the presence of the pharmaceutical chemicals. Among the weight loss supplements, 15.3% (95% CI: 9.2–21.4) contained undeclared sibutramine, 13.9% (95% CI: 8.01–19.7) contained undeclared phenolphthalein, and 5.1% (95% CI: 1.4–8.8) contained undeclared fluoxetine. Amongst all weight loss supplements, 17.5% (95% CI: 11.07–24) contained significant concentrations of either sibutramine, phenolphthalein, or fluoxetine. Whilst weight loss herbal supplements offered for sale in the UAE have relatively low percentages of undeclared pharmaceuticals, many people take several different supplements daily and may encounter quite high levels of combined exposure to toxic compounds.

## 1. Introduction

Excess weight or obesity is a serious public health problem for adults worldwide. In the 2018 global estimates by the World Health Organization, over 1.9 billion adults (39%) in 2017 were regarded as overweight [1]. It has been predicted that the 2020 figures will show an increase to 60% [2]. Obesity plays a central role in numerous health conditions, including heart disease, stroke, non-alcoholic fatty liver disease, erectile dysfunction, type II diabetes, high blood pressure, and cancer amongst others [2].

When weight loss supplements are adulterated, there are two general motivations for the adulteration. In economic adulteration, a cheaper ingredient replaces more expensive ingredients claimed on the label. In pharmaceutical adulteration, an active drug will be added to an allegedly botanical supplement, e.g., sibutramine might be added to a weight loss supplement that claims to be purely “natural.

Numerous people choose herbal products that claim to promote weight loss with the mistaken belief that such products are safer than normal pharmaceuticals. In fact, nutraceuticals may include ingredients on the FDA list of prohibited compounds, such as phenolphthalein and sibutramine [3]. Weight loss nutraceuticals may contain chemicals that have numerous side effects that can cause cancer, heart failure, and other potentially fatal outcomes [4]. Furthermore, these products sometimes contain stimulants, e.g., sibutramine or caffeine, that can have numerous side effects, including depression, insomnia, thoughts of suicide, and other fatal problems [5]. For these reasons, these products should be avoided by individuals who suffer from anxiety, panic attacks, hypertension, or heart conditions [4].

The last few years have seen a significant increase in the number of dietary supplements purchased. Unethical manufacturers or distributors purposely add pharmaceuticals to herbal dietary supplements to create instant pharmacological reactions or boost the biological action of the supplement [6,7]. Some of these products can contain a multiplicity of analogues or adulterants. Dietary supplements that have illegal additives of pharmaceutical drugs or analogues have additional health risks, and customers may not be aware of what they are taking [8,9,10,11,12].

Such additives pose a significant risk to public health, particularly long-term consumption [2,13]. Synthetic substances and/or prescription drug analogues in herbal weight loss products are concerning for all global health agencies [14,15,16]. Due to this, the WHO [9], Food and Drug Administration (FDA), and European Medicines Agency [10] have promulgated guidelines for the ways in which herbal medicines can be used safely and appropriately [17,18].

Another problem with these adulterated supplements is that they are often marketed and sold via the Internet or social media, where they can avoid strict controls and do not necessarily have to satisfy safety/control testing prior to marketing and selling [4].

Numerous stakeholders have issued reports on many occasions where herbal weight loss products that have been adulterated [19,20,21]. Amongst the undeclared active pharmaceutical ingredients (APIs) found in herbal products are lorcaserin, fluoxetine, phenolphthalein, sildenafil, and sibutramine [22].

In Korea, chlorosibutramine (an analogue of sibutramine) has been found to be added to weight loss products [19]. In an investigation of weight loss supplements sold in Brazil to look for undeclared APIs, Neves and Caldas [23] found fenproporex, phenolphthalein, sibutramine, and amfepramone. Wang et al. [24] found phenolphthalein, N-mono-desmethylsibutramine, and sibutramine in herbal weight loss products. These results demonstrate that consumers could, without knowing, be consuming products that control different levels of prescription drugs, controlled substances, and/or active ingredients that have not been thoroughly tested or researched. Such products can cause serious damage to a person’s health.

In the UAE regulatory regime, the manufacturers of dietary supplements must supply evidence that their product manufacturing environment guarantees their products are safe and pure; any new product must be tested in municipal laboratories. Nevertheless, despite these safeguards, there have been cases where microorganism-contaminated dietary supplements have gained approval and been offered for sale [25,26,27].

The Dubai Municipality has 11 circulars on its webpages that worn against the trade, sale, or use of weight loss products that have not been granted a license by the relevant authority and that may contain ingredients that are banned across the world; these ingredients could have an adverse impact on the health and safety of consumers [28,29].

With the globalization and increasing value of the market for weight loss supplements, and with the possible dangers to public health of the inadvertent ingestion of unmeasured quantities of pharmaceuticals, cases have been rising of individuals having an adverse reaction to weight loss supplements and even suffering mortality as a result of the products being contaminated or adulterated rather than a reaction to the ingredients claimed on the product. In certain instances, this is a result of manufacturers intentionally defrauding the public by producing a poor-quality product, using sophisticated techniques to circumvent regulatory and oversight procedures.

In light of these facts and due to the scarcity of scientific studies in this field in the UAE, this study hypothesized that weight loss herbal supplements offered for sale in the UAE have relatively considerable percentages of undeclared pharmaceuticals. This observational retrospective prevalence study is an essential investigation and quantification of illegally added pharmaceuticals or prescription medications, specifically fluoxetine, phenolphthalein, and sibutramine, in herbal weight loss supplements offered for sale in the UAE. The findings will help to demonstrate the most common ingredients in weight loss supplements offered for sale in the UAE, to show whether extant regulations are being properly observed and to help the regulatory bodies in developing a risk assessment module for the safety of weight loss supplements

## 2. Results

### 2.1. Sample Description

Table 1 shows the sample baseline characteristics of the weight loss supplements. In this case, 137 weight loss supplements were collected and analyzed in this study. The dosage forms of the weight loss supplement were 87 (63.5%) capsules, 31 (22.6%) tablets, and 19 (13.9%) teabags. Three samples (2.2%) were made in Canada, 67 (48.9%) were made in the USA, 22 (16.1%) were made in the European Union, seven (5.1%) were made in China, 6 (4.4%) were made in Malaysia, four (2.9%) were made in India, four (2.9%) were made in the UAE, and 24 (17.5%) did not have a declared country of origin.

### 2.2. Assessment of Hidden Prescription Drugs and Chemicals in the Weight Loss Supplements

Table 2 summarizes the number of test results in which the measured prescription drugs and chemicals were below the limit of detection (LOD). Among the weight loss supplements, 15.3% (95% CI: 9.2–21.4) contained undeclared sibutramine, 13.9% (95% CI: 8.01–19.7) contained undeclared phenolphthalein, and 5.1% (95% CI: 1.4–8.8) contained undeclared fluoxetine. Of the 137 weight loss supplements, seven (5.1%) included one undeclared prescription drug/chemical, 11 (8%) included two undeclared prescription drugs/chemicals, and six (4.4%) included three undeclared prescription drugs/chemicals. Amongst all of the weight loss supplements, 17.5% (95% CI: 11.07–24) contained significant concentrations of either sibutramine, phenolphthalein, or fluoxetine (Table 3). The prevalence of undeclared prescription drugs/chemicals in these weight loss supplements categorized by the sample characteristics of each sample is provided in Table 4. Supplementary Materials shows the Chromatograms for some analyzed weight loss supplements (Supplementary Figure S1).

### 2.3. Comparison of Undeclared Prescription Drugs/Chemicals in Weight Loss Supplements According to Sample Characteristics

Table 5 presents the distribution of undeclared prescription drugs/chemicals according to the sample characteristics. The table also provides the estimates with the *p*-values. These *p*-values were obtained from the results of the chi-square and Fisher’s exact tests. There was a statistical association between the labelled country of origin and the undeclared prescription drug/chemical (*p* < 0.001). An undeclared prescription drug/chemical was more prevalent in samples with an undeclared country of origin (66.7%) than in the samples with a clear country of origin (7.1%). However, there was no statistically significant association between dosage form and an undeclared prescription drug/chemical (*p* = 0.178).

## 3. Discussion

Weight loss nutraceuticals have become increasingly popular and more widely available, so there has been a rise in the number of adulterated products containing pharmaceutical chemicals or prescription drugs. Such contaminated products are a global problem, and they pose a health risk to those who consume them. The primary aim of this research was to investigate whether nutraceuticals offered for weight loss in the UAE are adulterated with pharmaceutical chemicals or prescription drugs.

This research analyzed 137 nutraceuticals sold as weight loss products; 17.5% were shown to have adulterations of significant quantities of one of the three drugs we were looking for. This level of adulteration is lower than that found in past research. In a study of 160 herbal weight loss supplements, Hachem et al. [22] revealed that over 50% had been adulterated with one of six different pharmaceutical chemicals. Research by the FDA of the USA showed that 572 dietary supplements were found to have been adulterated using pharmaceutical chemicals not listed as ingredients (228 of these were sold as weight loss products) in the years 2007 to 2014 [30]. Research carried out in Iran found that 72% of 61 products sold for weight loss contained pharmaceutical chemicals [31]. Research in Korea found nine separate additives in 76 out of 370 samples [32].

The fact that such a comparatively low rate of adulteration with pharmaceutical chemicals has been found in weight loss nutraceuticals offered for sale in the UAE may be attributed to the regulations in the country, with municipalities and health regulators insisting that every dietary supplement sold must participate in registration to prove that they are efficacious, of acceptable quality, and safe.

Sibutramine was the most widely used adulterant in the weight loss products tested, followed by phenolphthalein and fluoxetine. Sibutramine was found in 21 samples at varying levels (0.14–16,823.3 mg/kg). These findings are consistent with past research, where sibutramine was found to be the most frequent illegal additive in herbal weight loss preparations [19,32,33,34,35,36,37,38,39]. Sibutramine is added to dietary supplements promoted for weight loss because it exploits noradrenaline and serotonin reuptake inhibitors, leading to increases in the synaptic concentration of these neurotransmitters and subsequently activating the α-adrenoceptors, β-adrenoceptors, and serotonin receptors. These chemical effects cause people to feel more sated and burn more energy, which leads to a drop in body weight [34]. Amongst the damaging side effects of sibutramine are hepatitis, psychosis, arrhythmia, and additional cardiovascular conditions [40]. People who take herbal slimming preparations that have undeclared sibutramine may experience several side effects, from headaches to serious cardiovascular disturbance, depending on the amount of drug ingested [41,42,43].

The second most frequent illegal additive in herbal weight loss products in this research was the laxative phenolphthalein, which was found in 19 samples at levels of 1.9–13,218.8 mg/kg. This drug is often found alongside sibutramine as an illegal adulterant to weight loss supplements. These findings are consistent with the results of many other studies [35,36,44,45]. People who take herbal slimming preparations that contain undeclared phenolphthalein may face an increased risk of cancer [35,36,44].

The third most frequent illegal additive to have weight loss products in this research was fluoxetine. This was found in seven samples at levels between 12 and 193.97 mg/kg. This is in line with the results of an Iranian study [46].

It is a matter of serious concern that this research found six samples (4.4%) containing undeclared pharmaceutical chemicals and 11 (8%) containing more than one undeclared pharmaceutical chemical. There is potential for worry about the misuse of these diet products, as users could believe that ingesting more could accelerate the weight loss process, and this could cause addiction or toxic overdose. Potentially fatal results could occur from just one dose of a product that contains these chemical additives, and because consumers may believe the products’ claims to be safe, the consumer might take additional doses, which could result in extreme toxicity [47,48].

Even at a low dose, these pharmaceutical additives could be toxic and potentially fatal when taken without proper medical supervision. Matters could be made worse if the person taking the drug is also taking other prescription medication [33,49]. Patients might suffer side effects due to the pharmaceutical adulterants reacting with each other, with other drugs the patient is taking, or with the herbal constituents of the product [50,51].

Consuming excessive amounts of weight loss supplements that have been adulterated with pharmaceuticals creates serious health risks for patients, as it cannot be known what quantity of undeclared pharmaceutical chemicals the product contains [19]. To protect public health and to reinforce legal provisions, it is essential that weight loss dietary supplements should be effectively screened for undeclared pharmaceutical chemicals [52].

The results of this research have shown that it is more likely for a supplement to contain undeclared prescription drugs or chemicals when no country of origin is listed on the product in comparison to those where the country of origin is stated.

There is a necessity for consumers to be made aware of the potentially fatal results of taking illegal nutraceuticals, which are not subjected to regulatory regimes and do not require prescriptions. There should be robust laws developed that ban such illegal products from being sold, marketed, or advertised on television. Last, a reliable method should be employed to detect counterfeit supplements and any adulterants, e.g., simple spectrophotometric analysis and near-infrared spectrophotometry [53,54]. Several novel portable devices to identify drug adulterants are now available [54]. Additional research should be undertaken regarding the ways these devices can be optimized, validated, and modified to assist in the detection of adulterated and counterfeit products [44].

One reason for the adulteration of supplements being hard to regulate is that there are frequent disagreements between regulators, physicians, and manufacturers as to the amount of quality testing required for supplements. The World Health Organization’s Strategy on Traditional Medicines 2014–2023 [55] makes it clear that quality controls are essential for the manufacture of supplements. Botanical extracts and blends offer specific challenges in the detection of misidentified ingredients for contaminants. Both biological and chemical adulterants and contaminants, as they appear in supplements, offer a challenge. With some types of supplements, e.g., those for weight loss, enhanced sexual performance, and enhanced athletic performance, it is especially common to find undeclared extraneous/synthetic substances being added deliberately to cause confusion for analysts; sometimes active synthetic drugs may be added [56].

## 4. Materials and Methods

### 4.1. Chemicals

**Figure 1:**
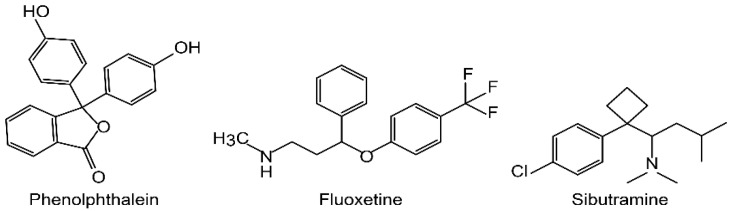
Chemical structure of fluoxetine, phenolphthalein, and sibutramine.

Sibutramine hydrochloride monohydrate (CAS#125494-59-9), fluoxetine (CAS# 347 54910-89-3), and phenolphthalein (CAS#77-09-8) (Figure 1) Dr. Ehrenstorfer, U.K. Sibutramine-d6 (CAS#1216544-25-0) and fluoxetine-d5 (CAS#1173020-43-3) from Dr. Ehrenstorfer, U.K were used as internal standards (ISTD). Acetonitrile, ammonium formate, formic acid and methanol were HPLC grade (Merck, Kenilworth, NJ, USA), and the water was deionized (Merck Milli-Q water purification system, Kenilworth, NJ, USA).

### 4.2. Sample Collection

We found a variety of outlets for weight loss nutraceuticals through searches of local business directories that hold information on healthcare shops, nutrition shops, para-pharmacies, and pharmacies in every emirate of the UAE. We found 1500 such outlets and created a sampling framework on an Excel spreadsheet containing the business name, location, email address, and telephone number. We then created a research sample employing a basic random selection procedure using the ID numbers we had assigned each business, with stratification for type and location. For all chosen locations, random selection was made of a single package of all weight loss nutraceuticals designed for oral use, regardless of where they were manufactured. All items were assigned a code reference number to avoid duplication in samples and to allow for tracking. We recorded details for each sample, including which part of the shop the product was found in, the manufacturer’s recommended dosage, the product size/volume, the dosage form, the batch number, the barcode, the category and subcategory, the name of the product, the nation of origin/manufacture, and the brand name. If more than one outlet offered identical products for sale (same name, manufacturer, formula, size/volume, barcode), we tested the sample that was obtained first and returned the others. In the instance when two products had identical names but came from different manufacturers or were offered in different forms (e.g., one liquid and one tablet), we put both products into the testing regime. All products were sent to the laboratory to be tested the day they were collected.

### 4.3. Sample Treatment

Tablets and capsules were homogenized by making fine powders using a mortar, pestle, and grinder. The homogenized material was then used for sample preparation. Powers and liquids were homogenized by stirring with a spatula or glass rod. The homogenized material was then used for sample preparation. Samples in the form of herbal tea-like leaves and roots were homogenized using a blender. The homogenized material was then used for sample preparation. 

Approximately 0.5 g of each food, dietary supplement, and health supplement as a homogenous sample were added into a 20 mL stoppered flask, with 400 µL of ISTD intermediate solution mix (1 ppm) and 15 mL of water:acetonitrile (20:80, *v/v*), sonicated to complete disintegration, cooled to room temperature, and diluted to the appropriate volume with diluent. The two phases were separated by centrifugation (6000 rpm), and then the supernatant solution was filtered through a 0.2 µm nylon syringe filter and put into an HPLC autosampler vial.

### 4.4. RP-HPLC-MS/MS Analysis

The reverse-phase liquid chromatography–high resolution mass spectrometry/mass spectrometry (RP-HPLC-MS/MS) analysis was performed with the Agilent 1260 infinity series 6420 Triple Quad MS system equipped with a Kinetex XB-C18 (100 Å, 50 × 2.1 mm) column. The mobile phases were A (3 mM ammonium formate + 0.1% formic acid in water) and B (0.1% formic acid in acetonitrile) at the gradient program is showed in Table 6. Chromatographic separation was achieved with gradient elution (5 μL of the sample was injected into the chromatographic system) at a flow rate of 0.25 mL/min and column temperature of 50 °C. The peaks of the determined components were identified by their mass and by comparing their retention time with that of standards, and the run time was 20 min (Table 7).

### 4.5. Validation Methodology

The method was fully validated according to the ICH (International Conference on Harmonization) guidelines by determination of linearity, precision, accuracy, limit of detection (LOD) and limit of quantification (LOQ). The selectivity of the method was proven with the chromatographic peak resolution obtained between sibutramine, fluoxetine and phenolphthalein. Calibration standards of sibutramine, fluoxetine, and phenolphthalein at concentrations of 3.0, 5.0, 10.0, 25.0, 50.0, and 100.0 µg/kg and internal standards of sibutramine-d6 and fluoxetine-d5 at a concentration of 20.0 µg/kg, which were added to the blank matrix (analyte free matrix). Spiked blank samples (analyte free matrix) were extracted into a 20 mL stoppered flask and analyzed following previously described sample preparation procedures. The solutions were stored in an amber-colored glass vial at −20 °C for long-term storage. The linearity of the method was tested in the range of 3.0–100.0 µg/kg with a correlation coefficient value greater than 0.995.

The limit of detection was determined on analyte-free samples (herbal tea, energy drink and fish oil capsules) with a signal-to-noise ratio of at least 3:1. The limit of quantification (LOQ) was estimated based on a signal-to-noise ratio of at least 10. The LOD and LOQ of the method were tested in the range of 40.0–120.0 µg/kg. The limits of the method were 10.0 µg/kg and 120.0 µg/kg for the LOD and LOQ, respectively.

The repeatability test for retention time (RT) and peak area was carried out by injecting the standard mixtures of 3 analytes with ISTD at concentrations of 3.0 µg/kg, 10 µg/kg and 80 µg/kg 6 times a day. The relative standard deviation (RSD) of intraday precision for RT and peak area was 0.42% and 6.93%, respectively.

The accuracy and precision procedure was demonstrated by spiking 6 individual solutions at concentrations ranging from LOQ to medium and high levels from the calibration concentrations. In this method validation, sibutramine, fluoxetine and phenol-phthalein were spiked at 3.0 µg/kg, 10 µg/kg and 80 µg/kg in analyte-free products such as herbal tea, energy drink and fish oil capsules were prepared and analyzed for each of the six spike levels. The %RSD and % Recoveries found as mentioned in Table 8.

Moreover, the reproducibility test was performed by injecting the standard mixtures of 3 analytes with ISTD at concentrations of 3.0 µg/kg, 10 µg/kg and 80 µg/kg 6 times for 2 days in analyte-free products such as herbal tea, energy drink and fish oil capsules were prepared and analyzed for each of the six spike levels. The %RSD and % Recoveries found as mentioned in Table 9.

To the quality control and quality assurance (QC/QA), 10 mg of sibutramine, fluoxetine, and phenolphthalein were transferred in methanol into a 10 mL volumetric flask to achieve a concentration of 1000 mg/L, and serial dilutions to 20 µg/L with methanol were made. This stock solution was stored in an amber-colored glass vial at −20 °C. The quality control of standard was evaluated with 10 µg/L concentration of the standard prepared separately from the different/same LOT, and the recovery was within 90–110%. For the quality control of samples, an analyte-free matrix was spiked at a concentration of 10 µg/kg, and the recovery was within 80–120%. To study the duplicate sample preparation, unknown samples were taken in duplicate, and the variation was not more than 10%. To evaluate the spiked sample preparation, unknown samples were spiked at a concentration of 10 µg/kg, obtaining a recovery within 80–120%. To check the standards, the same standard 10 µg/kg preparation was injected at the end of the sequence, and the recovery was within 90–110%.

Sibutramine, fluoxetine, and phenolphthalein quantifications were performed using the ratio of the intensity of the two major fragment ions. The peak area ratio (PAR) for each working solution by dividing the peak area of the sibutramine and fluoxetine (AREAsibutramine and fluoxetine) by the peak area of the internal standard (AREAIS) were calculated. Calibration curves from the PAR and the concentration of the standard solutions were constructed. The concentrations of sibutramine, fluoxetine, and phenolphthalein from the unknown samples were calculated by using the following formula: Concentration (mg/kg) = Instrument Con. (µg/kg) × Make up volume (mL) × Dilution Weight of sample (g) × 1000.

### 4.6. Statistical Analysis

Data analysis was performed using SPSS version 26 Chicago, IL, USA. The qualitative variables were displayed using frequencies and percentages. The incidence of undeclared pharmaceutical chemicals in the weight loss supplements was defined as the proportion of the products that contained significant concentrations of either sibutramine, phenolphthalein, or fluoxetine. For each weight loss supplement containing pharmaceutical chemicals, an estimate of the daily intake dose of each pharmaceutical chemical was calculated as the test concentration of the pharmaceutical chemical (mg/kg) and the manufacturer’s daily instructions for use (g). The Chi-square and Fisher exact tests were used to explore the factors linked to adulteration behaviors. *p*-values < 0.05 and a 95% confidence interval were set as the criteria for statistical significance.

## 5. Conclusions

While weight loss herbal supplements offered for sale in the UAE have relatively low percentages of undeclared pharmaceuticals, many people take several different supplements daily and may face quite high levels of combined toxic exposure. Information regarding the dangers presented by such products should be made available to healthcare workers, including practicing physicians, pharmacists, and nutritionists. In addition, more quality and safety checks should be undertaken on these sorts of products and greater controls should be introduced in terms of regulatory regimes, additional research, more education, and better reporting of undesirable outcomes.

## Figures and Tables

**Table 1:** Number and percentages of sample baseline characteristics (*n* = 137).

Characteristics	Groups	Frequency	Percentage
Dosage form	Capsule	87	63.5%
	Tablet	31	22.6%
	Tea bags	19	13.9%
Country of origin	Canada	3	2.2%
	USA	67	48.9%
	European Union	22	16.1%
	China	7	5.1%
	Malaysia	6	4.4%
	India	4	2.9%
	UAE	4	2.9%
	Undeclared	24	17.5%

**Table 2:** Distribution of weight loss supplements that contained the tested prescription drugs and chemicals below the limit of detection (*n* = 137).

Prescription Drug/Chemical	Number of Products with Prescription Drug/Chemical under the LOD	
	Frequency	Percentage
sibutramine	116	84.7%
phenolphthalein	118	86.1%
fluoxetine	130	94.95

Abbreviation: LOD, limit of detection = 10 μg/kg.

**Table 3:** Estimation of hidden prescription drugs and chemicals in weight loss supplements.

Prescription Drug/Chemical	The Proportion of Weight Loss Supplements That Contained Hidden Prescription Drugs/Chemicals			
	N	%	95% CI	
			Lower	Upper
sibutramine	21	15.3%	9.2	21.4
phenolphthalein	19	13.9%	8.01	19.7
fluoxetine	7	5.1%	1.4	8.8

Abbreviation: 95% CI: confidence interval, N: number of sample.

**Table 4:** List of tested weight loss supplement products according to the prescription drugs/chemicals and sample characteristics.

Sample Characteristics			Estimated Concentration (mg/kg)			Daily Dose (mg/day)			Number of Hidden Chemicals
Sample Code	Dosage Form	Country of Origin	Sibutramine	Phenolphthalein	Fluoxetine	Sibutramine Daily Dose	Phenolphthalein Daily Dose	Fluoxetine Daily Dose	N
1	Tablet	Undeclared	259	ND	ND	5.18	ND	ND	1
2	Tablet	China	258.8	ND	ND	0.11	ND	ND	1
3	Capsule	USA	29.06	117.07	ND	0.02	0.07	ND	2
4	Capsule	Undeclared	ND	199.5	193.97	ND	0.07	0.06	2
5	Capsule	Undeclared	172	183.48	ND	0.32	0.34	ND	2
6	Capsule	Undeclared	214.86	173.05	ND	0.11	0.09	ND	2
7	Capsule	Malaysia	101.36	146.79	14.42	0.09	0.13	0.01	3
8	Capsule	China	213.49	235.02	ND	111.01	122.21	ND	2
9	Capsule	USA	ND	163.1	ND	ND	0.91	ND	1
10	Capsule	Malaysia	ND	193.75	ND	ND	0.08	ND	1
11	Capsule	EU	44.75	202.92	12	0.17	0.78	46.08	3
12	Capsule	China	98.95	163.55	ND	0.05	0.09	ND	2
13	Capsule	Undeclared	16.88	ND	ND	0.01	ND	ND	1
14	Tea bags	Undeclared	10.63	ND	ND	0.15	ND	ND	1
15	Tablet	Undeclared	0.14	1.9	ND	0	0	ND	2
16	Capsule	Undeclared	16,823.3	13,218.8	34	11.77	9.25	8.24	3
17	Capsule	Undeclared	121.7	ND	ND	0.12	ND	ND	1
18	Capsule	Undeclared	99.5	345	ND	382.08	1324.8	ND	2
19	Capsule	Undeclared	130	189	ND	72.02	104.71	ND	2
20	Tablet	Undeclared	78	213	172	49.76	135.89	109.74	3
21	Tablet	Undeclared	123	245	ND	170.77	340.16	ND	2
22	Tablet	Undeclared	1237	234	ND	1355.75	256.46	ND	2
23	Tablet	Undeclared	16,823.3	123	57	11,776.31	86.1	39.9	3
24	Tablet	Undeclared	232	289	45	226.9	282.64	44.01	3

Notes: The daily dose was calculated by using the test concentration of each chemical (mg/kg) and the manufacturer’s advised daily adult dose (g). ND, not detected.

**Table 5:** Comparison of hidden prescription drugs and chemicals according to sample characteristics.

Characteristic	Group	Hidden Prescription Drugs and Chemicals		
		N	%	*p*-Value
Dosage form	Capsule	15	17.2%	0.178
	Tablet	8	25.8%	
	Tea bags	1	5.3%	
Country of origin	Declared	8	7.1%	<0.001
	Undeclared	16	66.7%	

*p*-values reported above are for comparisons between variable level “category-levels” using the Chi-square and Fisher exact tests. *p*-values less than 0.05 were considered statistically significant.

**Table 6:** Gradient program and MS conditions: Source parameters.

Gradient Program			MS Conditions-Source Parameters
**Time**	**% Mobile Phase A**	**% Mobile Phase B**	Ion Source: ESI Scan Type: MRM Div valve: To MS Delta EMV(+): 300 Gas Temp: 350 °C N2 Gas flow: 10 L/min Nebulizer: 50 psi Capillary Voltage: 4000 V (Positive and Negative) Chromatogram: TIC
0.0	65.0	35.0	
1.0	65.0	35.0	
7.0	10.0	90.0	
7.5	10.0	90.0	
8.0	65.0	35.0	
16.0	65.0	35.0	

MS: mass spectrometry.

**Table 7:** Acquisition parameters.

Compound Name	Precursor Ion	MS1 Res	Product Ion	MS2 Res	Dwell	FV	CE	CAV	Polarity
phenolphthalein	319.3	Wide	224.9 *	Unit	200	139	16	7	Positive
		Wide	141	Unit	200	139	40	7	Positive
sibutramine	280.9	Wide	139	Unit	200	96	8	7	Positive
		Wide	125 *	Unit	200	96	28	7	Positive
fluoxetine	310.1	Wide	117	Unit	200	96	50	7	Positive
		Wide	148 *	Unit	200	96	10	7	Positive
sibutramine-d6	286.9	Wide	125	Unit	200	101	28	7	Positive
fluoxetine-d5	316.2	Wide	154.1	Unit	200	70	2	7	Positive

* Product ions were used for quantitation, MS: mass spectrometry, Dwell: Dwell time, FV: fragmentor voltage, CE: collisional energy, CAV: cell accelerator voltage.

**Table 8:** Accuracy and Precision on day 1.

Matrix Name	% RSD of RT	Spike at 3.0 µg/kg		Spike at 10 µg/kg		Spike at 80 µg/kg	
		% RSD	% Recovery	% RSD	% Recovery	% RSD	% Recovery
Herbal Tea	0.4	7.3	83.6	6.1	87.9	5.3	93.5
Energy Drink	0.9	5.4	86.7	4.5	91.4	3.2	94.9
Fish oil capsules	1.5	8.8	83.9	7.6	88.5	5.0	90.6

RSD: relative standard deviation, RT: retention time.

**Table 9:** Accuracy and Precision on day 2 (Reproducibility).

Matrix Name	% RSD of RT	Spike at 3.0 µg/kg		Spike at 10 µg/kg		Spike at 80 µg/kg	
		% RSD	% Recovery	% RSD	% Recovery	% RSD	% Recovery
Herbal Tea	0.38	8.6	82.5	7.2	88.3	4.8	92.1
Energy Drink	0.8	5.5	85.1	4.2	90.9	4.0	93.3
Fish oil capsules	1.3	7.6	82.7	6.0	86.4	5.3	92.6

## Data Availability

Data is available from the corresponding author upon reasonable request.

## Supplementary Materials

The following are available online, Figure S1: shows the Chromatograms for some analyzed weight loss supplements.

## Sample Availability

Samples of the compounds are available on request from the corresponding author.
