# Supplementary material for: Adulteration of Weight Loss Supplements by the Illegal Addition of Synthetic Pharmaceuticals
Source: Molecules. 2021 Nov 16;26(22):6903. doi: 10.3390/molecules26226903 (PMC8621677; doi:10.3390/molecules26226903)
Supplement: Supplementary file 1 [file molecules-26-06903-s001.zip › molecules-1337705-supplementary.pdf]

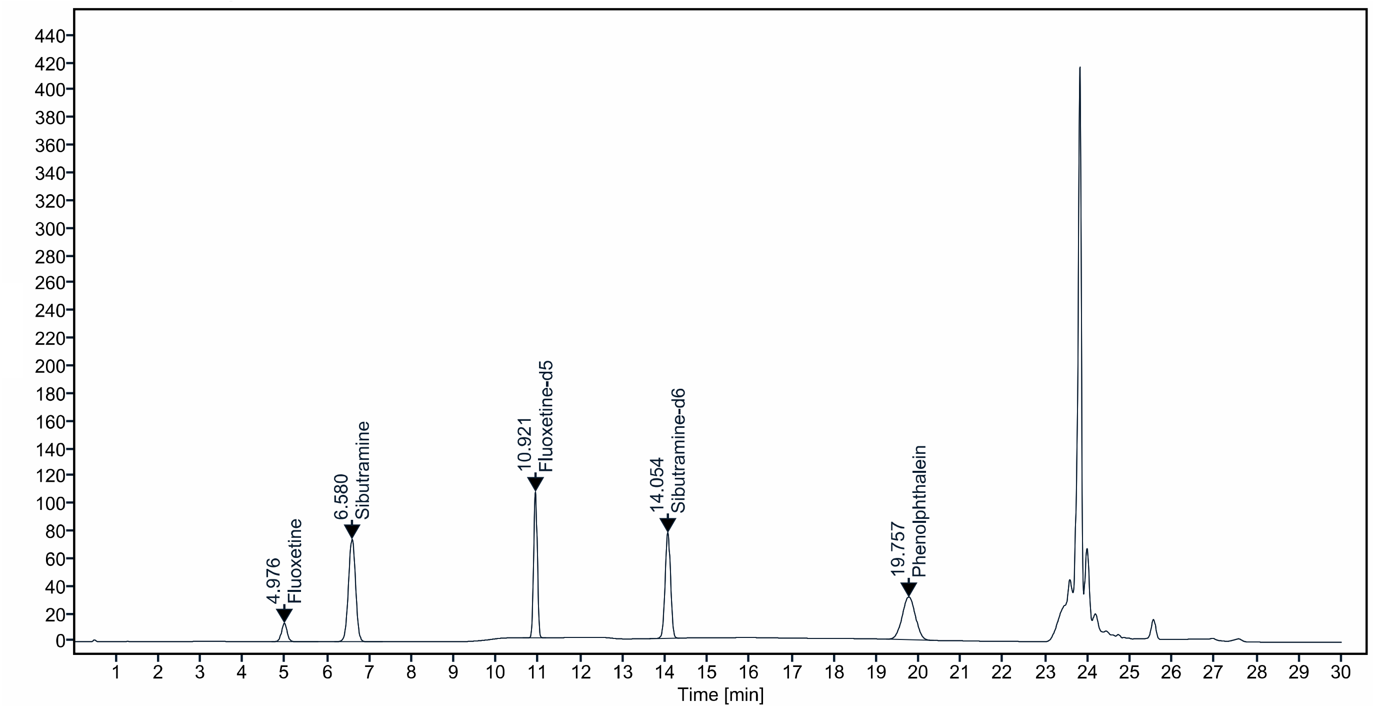


| Name | RT (min) | Area |
|---|---|---|
| Fluoxetine | 4.976 | 123.636 |
| Sibutramine | 6.580 | 877.825 |
| Fluoxetine-d5 | 10.921 | 637.695 |
| Sibutramine-d6 | 14.054 | 687.799 |
| Phenolphthalein | 19.757 | 679.323 |
| | Sum | 3006.2784 |

Figure S1. The chromatograms for some analyzed weight loss supplements.
